# Amoebic liver abscess in northern Sri Lanka: first report of immunological and molecular confirmation of aetiology

**DOI:** 10.1186/s13071-016-1950-2

**Published:** 2017-01-07

**Authors:** Selvam Kannathasan, Arumugam Murugananthan, Thirunavukarasu Kumanan, Devika Iddawala, Nilanthi Renuka de Silva, Nadarajah Rajeshkannan, Rashidul Haque

**Affiliations:** 1 Department of Pathology, Faculty of Medicine, University of Jaffna, Jaffna, Sri Lanka; 2 Department of Medicine, Faculty of Medicine, University of Jaffna, Jaffna, Sri Lanka; 3 Department of Parasitology, Faculty of Medicine, University of Peradeniya, Peradeniya, Sri Lanka; 4 Department of Parasitology, Faculty of Medicine, University of Kelaniya, Ragama, Sri Lanka; 5 Coomealla Health Aboriginal Corporation, Dareton, NSW Australia; 6 International Centre for Diarrhoeal Disease Research, Dhaka, Bangladesh

**Keywords:** Amoebic liver abscess, *Entamoeba histolytica*, Northern Sri Lanka

## Abstract

**Background:**

Since 1985, amoebic liver abscess (ALA) has been a public health problem in northern Sri Lanka. Clinicians arrive at a diagnosis based on clinical and ultrasonographic findings, which cannot differentiate pyogenic liver abscess (PLA) from ALA. As the treatment and outcome of the ALA and PLA differs, determining the etiological agent is crucial.

**Methods:**

All clinically diagnosed ALA patients admitted to the Teaching Hospital (TH) in Jaffna during the study period were included and the clinical features, haematological parameters, and ultrasound scanning findings were obtained. Aspirated pus, blood, and faecal samples from patients were also collected. Pus and faeces were examined microscopically for amoebae. Pus was cultured in Robinson’s medium for amoebae, and MacConkey and blood agar for bacterial growth. ELISA kits were used for immunodiagnosis of *Entamoeba histolytica* infection. DNA was extracted from selected pus samples and amplified using nested PCR and the purified product was sequenced.

**Results:**

From July 2012 to July 2015, 346 of 367 clinically diagnosed ALA patients admitted to Jaffna Teaching Hospital were enrolled in this study. Almost all patients (98.6%) were males with a history of heavy alcohol consumption (100%). The main clinical features were fever (100%), right hypochodric pain (100%), tender hepatomegaly (90%) and intercostal tenderness (60%). Most patients had leukocytosis (86.7%), elevated ESR (85.8%) and elevated alkaline phosphatase (72.3%). Most of the abscesses were in the right lobe (85.3%) and solitary (76.3%) in nature. Among the 221 (63.87%) drained abscesses, 93.2% were chocolate brown in colour with the mean volume of 41.22 ± 1.16 ml. Only four pus samples (2%) were positive for amoeba by culture and the rest of the pus and faecal samples were negative microscopically and by culture. Furthermore, all pus samples were negative for bacterial growth. Antibody against *E. histolytica* (99.7%) and the *E. histolytica* antigen were detected in the pus samples (100%). Moreover, PCR and sequencing confirmed these results.

**Conclusion:**

To our knowledge, this is the first report from Sri Lanka that provides immunological and molecular confirmation that *Entamoeba histolytica* is a common cause of liver abscesses in the region.

## Background

Amoebiasis caused by *Entamoeba histolytica*, is known to affect at least 50 million people around the world and is responsible for up to 100,000 deaths per annum [1, 2]. Amoebic liver abscess (ALA), the most common extra-intestinal manifestation of invasive amoebiasis, is associated with high morbidity and mortality if the condition is not diagnosed and treated promptly.

The first report of hepatic amoebiasis from Sri Lanka, then Ceylon, dates to 1821 [3]. Since then, reports describing cases from all over the island have been published [3–7]. After about 1975, while many cases of clinically diagnosed ALA have been reported from the northern part of Sri Lanka [8–11], no cases have yet been reported from the rest of the island. Since 1985, clinicians working at Jaffna Teaching Hospital have been treating suspected ALA patients who were usually referred either from private clinics or peripheral Government hospitals [8]. The diagnosis was mainly based on the patient’s history, clinical features and other investigations such as haematological parameters and ultrasonography.

However, it is well known that the clinical features of ALA (acute onset of fever, abdominal pain and hepatomegaly) are very similar to those of pyogenic liver abscess (PLA), as are the ultrasonographic features. Thus, it is difficult to differentiate ALA from PLA either clinically or by ultrasound imaging [12]. At the same time, differentiating between PLA and ALA is important because the treatment regimen is different.

Although antibody-based and molecular diagnostic assays are very sensitive and specific for the confirmation of ALA, the limited resources available at Jaffna Teaching Hospital has meant that none of the patients admitted there have had laboratory confirmation by a specific diagnostic assay. The need to confirm the aetiological agent causing clinically suspected ALA in Jaffna is further highlighted by the fact that most patients had no evidence of intestinal amoebiasis (dysentery).

Hence, this study was carried out to confirm the aetiological agent causing clinically suspected ALA as *Entamoeba histolytica*, with the aid of specific immunological and molecular tools, for the first time in Sri Lanka.

## Methods

### Study design

This study was carried out among all adult patients with suspected ALA admitted to the medical wards of Jaffna TH between July 2012 and July 2015.

### Data collection

Information on the clinical features, haematological parameters such as full blood count, liver function tests, erythrocyte sedimentation rate (ESR), co-morbid conditions, and ultrasound findings were collected from the bed head ticket (BHT) after obtaining permission from the respective clinicians.

### Sample collection

#### Collection of pus from the abscess

A pus sample was collected while ultrasound guided aspiration was carried out as part of the treatment and management procedure. The total volume and colour of the pus were recorded. The last portion of the pus was collected into a sterile bottle and immediately transported to the Laboratory at the Division of Parasitology, Department of Pathology, Faculty of Medicine, University of Jaffna for the direct microscopic examination of the smear and culture. An aliquot of pus was stored at -40 °C for further immunological and molecular investigations.

#### Collection of blood from patients and controls

A sample of 3 ml venous blood was collected from all patients under sterile conditions, before treatment commenced. Control samples were collected from 100 randomly selected, healthy individuals (university students) who had been living for more than 5 years in northern Sri Lanka. Serum was separated from the clotted blood sample by centrifugation at 3500× *rpm* /10 min and stored at -20 °C for further serological investigations.

#### Collection of faecal samples

Faecal samples were collected from each patient in sterile, dry containers and transported to the laboratory on the same day. This procedure was repeated for three consecutive days from each patient.

### Basic laboratory diagnosis of ALA

#### Microscopic demonstration of the parasite

Both wet smears and permanent smears stained with trichrome were examined for trophozoites or cysts of *E. histolytica* in the pus, faecal sample, and in cultures.

#### Culture of *Entamoeba* spp.

Parasite culture was performed as per the method described by Robinson [13]. Briefly, freshly collected pus and faecal samples were inoculated separately in Robinson medium and incubated at 37 °C for 48–72 h.

#### Culture of bacteria

A loop of fresh pus sample was inoculated directly onto the surface of pre-warmed MacConkey and blood agar plates and streaked. The inoculated plates were incubated aerobically at 37 °C. Plates were examined after 18–24 h of incubation for the presence of bacterial growth.

### Serological investigations

#### *Entamoeba histolytica* IgG ELISA

Serum samples stored at -20 °C were thawed and examined for circulating IgG antibody against *E. histolytica* using an AccuDiag™ *E. histolytica* IgG (Amoebiasis) ELISA kit (California, USA), as per the manufacturer’s instructions.

#### *Entamoeba histolytica* antigen detection ELISA

Pus and serum samples were examined for *E. histolytica* antigen using an *E. histolytica/E. dispar* antigen detection ELISA kit from Diagnostic Automation/Cortez Diagnostics, Inc. (California, USA), as per the manufacturer’s instructions.

### Molecular diagnosis

DNA was extracted from each pus sample using a QIAGEN stool mini kit (Maryland, USA), as per the manufacturer’s instructions.

A nested PCR was performed based on the serine-rich *E. histolytica* protein (SREHP) coding gene of the parasite [14]. Briefly, 2 μl of extracted genomic DNA was used in the primary PCR in a 25 μl reaction using the PCR master mix (Promega, Wisconsin, USA) and the primers, SREHP-5 (5′-GCT AGT CCT GAA AAG CTT GAA GAA GCT G-3′) and SREHP-3 (5′-GGA CTT GAT GCA GCA TCA AGG T-3′). For the secondary reaction, the same 25 μl reaction was prepared though 2 μl of a 1:50 dilution of the initial PCR product was used as template. Also, a second set of nested primers; nSREHP-5 (5′-TAT TAT TAT CGT TAT CTG AAC TAC TTC CTG-3′) and nSREHP-3 (5′-GAA GAT AAT GAA GAT GAT GAA GAT G-3′) was used.

The temperature cycling conditions for the primary PCR amplification included an initial denaturing step, carried out at 94 °C for 15 min. This was followed by 40 cycles of 94 °C for 1 min, 50 °C for 1.5 min and 72 °C for 2 min, with a final extension of 72 °C for 5 min. The secondary PCR used the same temperature cycling conditions, with one modification; an annealing temperature of 55 °C [14]. One representative PCR product was purified using commercially purchased QIAquick PCR Purification Kit (QIAGEN), according to the protocol of the manufacturer. The purified PCR product was sequenced bi-directionally using the ABI 3500 Dx capillary instrument (Life Technologies, California, USA) at the Institute of Biochemistry, Molecular Biology and Biotechnology (IBMBB), University of Colombo, Sri Lanka.

### Analysis of data

Data were analyzed using SPSS statistical software (Version 16). Sequences generated were subjected to BLAST searches against AmoebaDB (http://amoebadb.org/amoeba/).

## Results

### History and clinical presentation

A total of 346 suspected ALA patients were enrolled in this study from July 2012 to July 2015. Among these patients, almost all (98.6%) were males with a history of consumption of alcohol (100%), especially a local drink, consisting of the fermented sap of the Palmyrah palm (toddy). The predominant clinical features observed were fever, right hypochodric pain with or without abdominal pain (Table 1). All the patients had high swinging fever with the average temperature of 38.9 ± 0.5 °C and the majority (98.26%) complained of fever for more than 5 days. Further, all the patients complained of right hypochodric pain, and a small number of patients (10%) had pain at the right shoulder tip, whereas roughly half of them reported abdominal pain (46.8%). A few of them (27.5%) reported nausea and vomiting. A few reported (7.8%) diarrhoea within the last 6 months, but the diarrhoea they described was of the watery type. Other than 24 patients (6.9%) who identified themselves as diabetic, there was no other significant co-morbidity (Table 1).

**Table 1:** History, clinical presentation, haematological parameters and ultrasound findings of the clinically diagnosed amoebic liver abscess patients admitted to the Teaching Hospital, Jaffna

Variable	Category	Sub-category	Percentage (*n* = 346)	95% CI
History	Sex	Male	98.6	96.8–99.5
		Female	1.4	
	Age	31–40	31.8	27.0–36.1
		41–50	62.4	57.2–67.4
		51–60	5.8	
	Alcohol consumption		100.0	
	Fever		100.0	
Clinical findings	Right hypochodric pain		100.0	
	Abdominal pain		46.8	41.6–52.1
	Nausea and vomiting		27.5	22.9–32.3
	Diabetes		6.9	4.6–10.0
Haematological parameters	Total white cell count	Not performed	6.1	
		Slightly high	7.2	
		Markedly elevated	86.7	82.8–90.0
	Erythrocyte sedimentation rate	Not performed	5.8	
		Slightly high	8.4	
		Markedly elevated	85.8	81.9–89.2
	Aspartate transaminase	Not performed	16.2	
		Normal	32.4	
		Slightly high	13.6	
		Markedly elevated	37.8	32.9–43.1
	Alkaline phosphatase	Not performed	15.9	
		Normal	0.9	
		Slightly High	11.0	
		Markedly elevated	72.3	67.4–76.8
Ultrasound findings	Location of the abscesses	Right lobe	85.3	81.2–88.7
		Left lobe	14.7	
	Number of abscesses	solitary	76.3	
		multiple	23.7	

#### Haematological parameters

Most patients had leucocytosis (86.7%), elevated ESR (85.8%) and elevated alkaline phosphatase (ALP) (72.3%). Elevated aspartate transaminase (AST/SGOT) was observed in some patients (37.8%) and slightly elevated in very few (13.6%) (Table 1).

#### Ultrasound findings

Most of the abscesses (85.3%) were found in the right lobe of the liver whereas 51 were found in the left lobe (Table 1). Most patients (76.3%) had a solitary abscess.

#### Details of abscess fluid

A total of 221 (63.87%) abscesses were drained within this 3-year period. The pus drained from almost all patients (93.2%) was chocolate brown in colour. The volume of pus varied, but more than 50 ml was drained from 133 abscesses. The average volume of pus drained from all abscesses was 41.22 ± 1.16 ml.

#### Microscopic examination and the culture of pus samples

Trophozoites of *E. histolytica* were not observed in any of 221 saline smears prepared from aspirated pus. However, four pus samples (2%) cultured in Robinson’s media were positive for *E. histolytica*. In addition, all 221 samples were negative for bacterial growth on MacConkey and blood agar.

#### Microscopic examination and the culture of faecal samples

Only 207 (59.8%) faecal samples were obtained from the patients. Among them, 30% provided three faecal samples, 40% provided two, and remainder gave only one sample. All 207 faecal samples were negative for *Entamoeba* spp. by microscopic examination and culture in Robinson’s media.

### Immunological diagnosis

Of 346 serum samples tested for *E. histolytica* circulating antigen, 344 were positive (99.4%). All 221 pus samples (100%) collected from patients who were sero-positive for *E. histolytica*, were positive for *E. histolytica* antigen (Table 2).

**Table 2:** Immunological findings of serum and pus samples of the ALA patient attended to the Teaching Hospital Jaffna

Sample	Antibody (IgG) positive rate			Antigen positive rate		
	Negative	Moderately positive	Highly positive	Negative	Moderately positive	Highly positive
Serum (N-346)	0.3% (1/346)	0	99.7% (345/346)	0.6% (2/346)	0	99.4% (344/346)
Pus (N-221)	Not performed	Not performed	Not performed	0	0	100.0% (346/346)

Among the 346 serum samples investigated, 345 were positive (99.7%) for IgG antibody against *E. histolytica.* Although the one sample which was negative for IgG antibody was positive for circulating *Entamoeba* antigen.

Moreover, *E. histolytica* IgG antibodies were negative in 100 serum samples collected from healthy individuals, suggesting that the sensitivity of the test is 100% (345/345) and the specificity is 99.01% (100/101). Further, the positive and negative predictive values of the test were 99.7% (345/346) and 100% (100/100), respectively (Table 3).

**Table 3:** Validity of the antibody test used for the serological diagnosis in our local context

	IgG antibody test ^a,b,c,d^		Total
	Positive	Negative	
Cases	345	1	346
Healthy individuals (University students)	0	100	100
Total	345	101	446

^a^Sensitivity = 100%; ^b^Specificity = 99.01%; ^c^Positive Predictive value = 99.7%; ^d^Negative Predictive Value = 100%

### PCR diagnosis

**Fig. 1:**
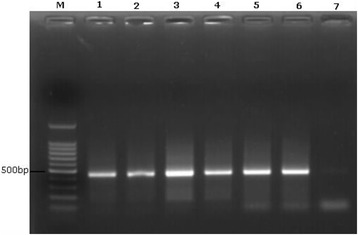
Results of nested PCR, amplified serine-rich protein coding gene (450 bp) of *Entamoeba histolytica*. Lane M: 100 bp ladder; Lanes 1–6: liver abscess aspirate samples; Lane 7: negative control

Nested PCR was performed on selected DNA samples extracted individually from the pus aspirated from 50 ALA patients. The targeted *Entamoeba* species DNA sequence was detected in all 50 pus samples (Fig. 1). The 450 bp sequence obtained from the PCR amplicon was subjected to NCBI BLASTn analysis (www.ncbi.nlm.nih.gov; GenBank KX377525) and comparison to sequences available on the Amoeba DB web site (http://amoebadb.org/amoeba/). The sequences generated in this study were 99% similar to corresponding gene sequences of serine-rich *E. histolytica* protein [SREHP].

## Discussion

This is the first report utilising immunological and molecular testing to confirm that *E. histolytica* is the aetiological agent of liver abscesses in northern Sri Lanka. Currently at the Jaffna TH, ALA is mainly diagnosed based on clinical findings (fever with right hypochondric and/or abdominal pain), history of heavy consumption of alcohol, followed by ultrasonography. This diagnosis is also supported based on a positive response to treatment with metronidazole.

In this study we examine 346 clinically diagnosed cases of ALA admitted to Jaffna TH. All patients complained of fever on admission. In addition, all had right hypochodric pain with or without abdominal pain. Similar presenting features have been reported from many other studies, conducted in Sri Lanka and elsewhere [3, 8–12, 15–24].

Findings on clinical examination were similar to those described previously in Sri Lanka and other Asian countries [15–17]. Haematological findings such as leukocytosis, elevated ESR and ALP were also comparable to those described previously [16–19, 22–27]. To facilitate the diagnosis, laboratory investigations play a major role. In our study, only 2% (4/221) of pus samples were positive in Robinson’s culture media. All pus samples and faecal samples were negative for the presence of *E. histolytica* microscopically (wet smears in saline as well as trichrome stained smears). Furthermore, all pus samples were negative for bacterial growth indicating that they were sterile and unlikely to be PLA. As the parasite resides in the periphery of the abscess wall, the probability of obtaining the amoeba in pus is very unlikely [28, 29]. Also, ALA may occur many months after the original intestinal infection is cleared. Hence, detecting the parasite in the faecal sample is also unlikely in the absence of concurrent dysentery [28]. Haque et al. reported that the sensitivity of microscopy for detecting amoebae in the pus of ALA as < 20% [29]. Other investigators report similar findings [12, 21, 23, 30].

Since the microscopic and culture methods available for detecting *E. histolytica* are less sensitive, immunological and molecular techniques are widely used. In Sri Lanka, these techniques have not been used so far to determine the causative agent of liver abscesses in northern Sri Lanka.

Although the detection of specific antibodies to *E. histolytica* in serum is thought to be highly sensitive for the diagnosis of ALA [31, 32], people with amoebic infection in endemic areas may have been repeatedly exposed to *E. histolytica* and remain asymptomatic. This situation makes the definitive diagnosis by antibody detection difficult because of the inability to distinguish a previous infection from a current infection. Combining clinical presentation and antigen detection with antibody detection will provide a definitive diagnosis of current infections.

In our study, 99.7% of serum samples were positive for IgG antibody against *E. histolytica*. These findings are comparable to those of others [33, 34]. Antigen detection ELISA has several significant advantages compared with other methods currently used for the diagnosis of amoebiasis such as microscopy, culture and even antibody detection tests [31]. The presence of detectable antigen in serum and pus indicates an ongoing infection [31]. In our study, 99.4% of serum and 100% of pus samples were positive for *E. histolytica* antigen. This provides immunological confirmation for the first time, that the clinical diagnosis of ALA by clinicians at Jaffna TH is well supported.

Many laboratories experience difficulties in diagnosing ALA using microscopy, faecal concentrates or culture-based methods and even using antibody detection in the serum in endemic areas. Hence, more sensitive and specific DNA-based detection methods have become popular as a solution to overcome these constraints [31, 35, 36]. Though successful use of PCR for the diagnosis of *E. histolytica* has been reported in many countries [37–40] no report has been published to date from Sri Lanka.

In our study, we targeted the serine-rich protein-coding gene which has been widely used elsewhere [14, 41–43]. By applying PCR and sequencing PCR amplicons in this study, we confirm *Entamoeba histolytica* as a common cause of liver abscesses in northern Sri Lanka.

## Conclusion

To our knowledge, this is the first study from Sri Lanka that provides immunological and molecular confirmation that *Entamoeba histolytica* is a common cause of clinically diagnosed liver abscess in northern Sri Lanka.

## Abbreviations

ALA: Amoebic liver abscess
ALP: Alkaline phosphatase
AST: Aspartate transaminase
BHT: Bed head ticket
ELISA: Enzyme-linked immunosorbent assay
ESR: Erythrocyte sedimentation rate
IgG: Immunoglobulin G
PLA: Pyogenic liver abscess
SREHP: Serine-rich *Entamoeba histolytica* protein
